# Adaptive diffusion kernel learning from biological networks for protein function prediction

**DOI:** 10.1186/1471-2105-9-162

**Published:** 2008-03-25

**Authors:** Liang Sun, Shuiwang Ji, Jieping Ye

**Affiliations:** 1 Center for Evolutionary Functional Genomics, The Biodesign Institute, Arizona State University, Tempe, AZ, USA; 2 Department of Computer Science and Engineering, Arizona State University, Tempe, AZ, USA

## Abstract

**Background:**

Machine-learning tools have gained considerable attention during the last few years for analyzing biological networks for protein function prediction. Kernel methods are suitable for learning from graph-based data such as biological networks, as they only require the abstraction of the similarities between objects into the kernel matrix. One key issue in kernel methods is the selection of a good kernel function. Diffusion kernels, the discretization of the familiar Gaussian kernel of Euclidean space, are commonly used for graph-based data.

**Results:**

In this paper, we address the issue of learning an optimal diffusion kernel, in the form of a convex combination of a set of pre-specified kernels constructed from biological networks, for protein function prediction. Most prior work on this kernel learning task focus on variants of the loss function based on Support Vector Machines (SVM). Their extensions to other loss functions such as the one based on Kullback-Leibler (KL) divergence, which is more suitable for mining biological networks, lead to expensive optimization problems. By exploiting the special structure of the diffusion kernel, we show that this KL divergence based kernel learning problem can be formulated as a simple optimization problem, which can then be solved efficiently. It is further extended to the multi-task case where we predict multiple functions of a protein simultaneously. We evaluate the efficiency and effectiveness of the proposed algorithms using two benchmark data sets.

**Conclusion:**

Results show that the performance of linearly combined diffusion kernel is better than every single candidate diffusion kernel. When the number of tasks is large, the algorithms based on multiple tasks are favored due to their competitive recognition performance and small computational costs.

## Background

Many types of genomic data can be represented as a graph (network), where the nodes represent genes or proteins, and edges may represent similarities between protein sequences, edges in a metabolic pathway, and physical interactions between proteins [1]. Machine learning tools have been commonly used to analyze biological networks for knowledge discovery and pattern analysis [2]. In this paper, we focus on learning from biological networks for protein function prediction. This problem has been studied extensively by using computational approaches recently [1]. Neighborhood-based methods [3,4] assign functions to proteins based on the most frequent functions within a neighborhood of the protein and they differ mainly in how the "neighborhood" of a protein is defined. More sophisticated prediction functions have been exploited in [5,6]. Methods based on network diffusion [7,8] view the protein network as a flow network and functions of proteins are diffused from annotated proteins to their neighbors in various ways. Other approaches for protein function annotation from biological networks include the graph-cut-based approaches [9,10] and those derived from the kernel methods [11-13].

Kernel methods are versatile tools for learning from graph-based data, as they only require the characterization of similarities between objects by the use of kernel trick [2,14]. Diffusion kernels [15], which can be considered as the discretization of the well-known Gaussian kernel of Euclidean space, are commonly used for graph-based data. In kernel methods, the information on the data is conveyed only in the kernel function, which uniquely determines the mapping of the original inputs onto a feature space. Thus, one of the central issues in kernel methods is the selection of a good kernel function for a specific problem at hand. A recent trend in kernel learning (selection) is to formulate it as convex programs, which lead to a globally optimal solution [16]. The idea of learning a linear combination of pre-specified kernels for Support Vector Machines (SVM) was originally proposed in [17] where this problem was formulated as semidefinite programs (SDP) and Quadratically Constrained Quadratic Programs (QCQP). In general, approaches based on learning a convex combination of kernels offer the additional advantage of facilitating heterogeneous data integration from different sources [18].

The objective functions for kernel learning used in [17] are performance measures for hard margin SVM, 1-norm soft margin SVM, and 2-norm soft margin SVM. An alternative criterion for kernel matrix learning is the Kullback-Leibler (KL) divergence [19] between the two zero-mean Gaussian distributions defined by the input and output kernel matrices [20]. One particularly appealing feature of the KL divergence criterion is that unlabeled (test) data can be integrated naturally into the training process, thereby improving generalizations. The formulations in [17] also use unlabeled data, but in a weak form by enforcing the trace magnitude of the kernel matrix including both training and test data in the constraint. Direct incorporation of unlabeled data by the formulations in [17] through the KL divergence criterion involves a matrix determinant term. The resulting formulation is a so-called *maximum-determinant problem *[21], which is a general framework that contains semidefinite programming (SDP) [16] as a special case. Although its theoretical soundness, experiences with semidefinite programming indicate that it is computationally expensive and thus can not be scaled to large-scale problems. The maximum-determinant problem is a more general framework than SDP and the path-following algorithms used to solve it is more expensive.

Diffusion kernels [15] capture the long-range relationships between vertices of graphs and are state-of-the-art for building kernels for graphs. In this paper, we focus on learning diffusion kernels constructed from biological networks, using the KL divergence criterion. In particular, we show that when the KL divergence criterion is used to optimize a convex combination of diffusion kernels with different parameters, the resulting optimization problem does not involve the matrix determinant term and thus can be solved by gradient descent methods. Previous studies [22,23] have shown that the removal of the matrix-determinant term in the KL divergence criterion has limited effect on its performance. When this modified criterion is used to learn a linear combination of diffusion kernels, the resulting optimization problem is convex and thus solutions by gradient descent methods are guaranteed to be globally optimal. A protein typically performs multiple functions. Most existing approaches formulate a separate task for each of the functions and they are learned independently. They decouple the functions of proteins and potentially compromise the performance as the functions of proteins are usually related. We show that our single-task kernel learning formulation based on the KL divergence criterion can be extended to the multi-task case by enforcing all tasks to share a common kernel. The resulting formulation leads to a single optimization problem, which learns multiple functions of proteins simultaneously. Experimental results show that this multiple-task kernel learning in a joint optimization framework keeps competitive prediction performance, while its computational cost is similar to that for a single task, thus dramatically reducing the time complexity.

## Methods

We study the problem of protein function prediction from biological networks, which are represented as graphs. For a graph G, the vertices represent proteins and edges characterize the relationship between proteins. In the following discussion, the vertex and edge sets are denoted as *V *and *E*, respectively. The total number of proteins in the network is *n *= |*V*|. The adjacency matrix *A *is used to denote the similarity between vertices where *A*_*i*,*j *_describes the similarity between vertices *v*_*i *_and *v*_*j*_. The functions of some proteins in the network are already known and the goal of protein function prediction is to infer the functions of unannotated proteins based on the functions of annotated proteins and the network topology. In particular, for a graph G = (*V*, *E*), the vertices in *V *can be partitioned into a training set and a test set. The functions of proteins in the training set are already known while those of proteins in the test set are unknown. Each edge in *E *reflects the local similarities between its ending vertices. The learning problem is to predict the functions of proteins in the test set based on the label information of training set and the topology of the graph.

### Background and Related Work

Kernel methods are particularly suitable for learning from graph-based data, as they only require the similarities between proteins to be encoded in the kernel matrix. In kernel methods, a symmetric function κ:X×X→R, where X denotes the input space, is called a kernel function if it satisfies the Mercer's condition [14]. When used for a finite number of samples in practice, this condition can be stated as follows: for any *x*_1_, ...,*x*_*n *_∈ X the *Gram *matrix *K *∈ ℝ^*n *× *n*^, defined by *K*_*ij *_= *κ*(*x*_*i*_, *x*_*j*_) is positive semidefinite. Any kernel function *κ *implicitly maps the input set X to a high-dimensional (possibly infinite) Hilbert space ${ℋ}_{\kappa }$ equipped with the inner product ${\left(\cdot ,\cdot \right)}_{{ℋ}_{\kappa }}$ through a mapping $\varphi_{\kappa }:X\to {ℋ}_{\kappa }$

(1) $$\kappa (x,z)={\left(\varphi_{\kappa }(x),\varphi_{\kappa }(z)\right)}_{{ℋ}_{\kappa }}.$$

The adjacency matrix *A *can't be directly used as a kernel matrix. First, the adjacency matrix contains the local similarity information only, which may not be effective for function prediction. Secondly, the adjacency matrix may not even be positive semidefinite. To derive a kernel matrix from the adjacency matrix, the idea of random walk and network diffusion has been used. The basic idea is to compute the global similarity between vertices *v*_*i *_and *v*_*j *_as the probability of reaching *v*_*j *_at some time point *T *when the random walker starts from *v*_*i*_. This idea is justified at least to some extent by observing that the random walker tends to meander around its origin as there is a larger number of paths of length |*T*| to its neighbors than to remote vertices [2].

To avoid some potential problems such as the choice of value for *T *and assurance of positive semidefiniteness for the kernel matrix, a random walk with an infinite number of infinitesimally small steps is used instead. It can be formally described as:

(2) $$K=\underset{s\to \infty }{\lim}{\left(I+\frac{\beta L}{s}\right)}^{s}=e^{\beta L},$$

where *β *is a parameter for controlling the extent of diffusion and *L *∈ ℝ^*n *× *n *^is the graph Laplacian matrix defined as

(3) *L *= diag(*Ae*) - *A*,

where *A *is the adjacency matrix, *e *is the vector of all ones, and diag(*Ae*) is a diagonal matrix with the diagonal entries being the corresponding row summation of the matrix *A*. It turns out that for any symmetric matrix *L*, *e*^*βL *^is always positive definite and thus can be used as a kernel matrix. The diffusion effect of such kernel can be explicitly seen when it is expanded as [2]:

(4) $$e^{\beta L}=I+\beta L+\frac{\beta^{2}}{2}L^{2}+\frac{\beta^{3}}{6}L^{3}+\cdots ,$$

where the local information encoded in *L *is continuously diffused by repeated multiplications. The parameter *β *in the diffusion kernel controls the extent of diffusion and it has a similar effect as the scaling parameter in Gaussian kernels. If the *β *is too small, the local information can not be diffused effectively, resulting in a kernel matrix that only captures local similarities. On the other hand, if it is too large, the neighborhood information will be lost. Furthermore, the optimal value for *β *is problem and data-dependent. Thus it is highly desirable to tune the *β *value adaptively from the data.

We approach the kernel tuning problem by learning an optimal kernel as a linear combination of pre-specified diffusion kernels constructed with different values of *β*. This is motivated from the work in [17] where the optimal kernel for SVM, in the form of a linear combination of pre-specified kernels, is learned based on the large margin criteria. In particular, the generalized performance measure based on 1-norm soft margin SVM used in [17] is

(5) $$\omega_{S1}(K)=\underset{\alpha :C\geq \alpha \geq 0,\alpha^{T}y=0}{\max}\{2\alpha^{T}e-\alpha^{T}G(K)\alpha \},$$

where *C *> 0 is the regularization parameter in SVM, *e *is the vector of all ones, *G*(*K*) is defined by *G*_*ij*_(*K*) = *k*(*x*_*i*_, *x*_*j*_)*y*_*i*_*y*_*j*_, and the *i*-th entry of *y *denoted as *y*_*i *_is the class label (1 or -1) of the *i*-th data point *x*_*i*_. Lanckriet *et al*. [17] showed that when the optimal kernel is restricted to the linear combination of the given *p *kernels *K*_1_, ..., *K*_*p*_, the kernel learning problem can be formulated as a semidefinite program. Furthermore, when the coefficients of the linear combination are constrained to be non-negative, the kernel learning problem can be formulated as a Quadratically Constrained Quadratic Program [16]. As was shown in [20], an alternative performance measure is the KL divergence between the two zero-mean Gaussian distributions associated with the input and output kernel matrices. We show that when this KL divergence criterion is used to learn a linear combination of diffusion kernels constructed with different values of *β*, the resulting optimization problem can be solved efficiently. We further show that it can be extended to the multiple-task case. Such integration of multiple tasks into one optimization problem can potentially exploit the complementary information among different tasks.

### Diffusion Kernel Learning: The Single-Task Case

We focus on learning an optimal kernel for a single task, which will then be extended to the multi-task case. The underlying idea is that the Laplacian matrix *L*, defined in Eq. (3), contains the connectivity information of all vertices in the graph. By adaptively tuning the kernel constructed from *L *on the training vertices, the entries corresponding to test vertices are expected to be tuned in some optimal way as well. To restrict the search space and improve the generalization ability, we focus on learning an optimal kernel as a linear combination of a set of diffusion kernels constructed with different values of *β*, indicating different extents of diffusion. In particular, we choose a sequence of values for *β *as *β*_1_, ...,*β*_*p*_, and the corresponding diffusion kernels can be constructed as

(6) $$\begin{array}{cc} K_{i}=e^{\beta_{i}L}, & i=1,\cdots ,p. \end{array}$$

We may assume that the kernels defined in Eq. (6) reflect our (weak) prior knowledge about the problem. The goal is to integrate the tuning of the coefficients into the learning process and the algorithm can adaptively select an optimal linear combination of the given kernels. Note that it is numerically favorable to normalize the kernels though this does not affect the results theoretically [14]. We normalize the kernels as follows:

(7) $${\tilde{K}}_{i}=\frac{e^{\beta_{i}L}}{\text{trace}(e^{\beta_{i}L})},$$

and the optimal kernel can be represented as

(8) $$K_{opt}=\sum_{i=1}^{p}\alpha_{i}{\tilde{K}}_{i}=\sum_{i=1}^{p}\alpha_{i}\frac{e^{\beta_{i}L}}{trace(e^{\beta_{i}L})},$$

for a set of non-negative coefficients ${\left\{\alpha_{i}\right\}}_{i=1}^{p}$

#### Kullback-Leibler Divergence Formulation

Kernel matrices are positive semidefinite and thus can be used as the covariance matrices for Gaussian distributions. It was shown in [20] that the kernel matrix can be learned by minimizing the Kullback-Leibler (KL) divergence between the zero-mean Gaussian distributions associated with the input and output kernel matrices. In this paper, we focus on learning the optimal coefficients *α*_*i *_from the data automatically based on minimizing this KL divergence criterion. As described in [20], the KL divergence between the zero-mean Gaussian distributions defined by the input kernel *K*_*x *_and output kernel *K*_*y *_can be expressed as

(9) $$\text{KL}(N_{y}|N_{x})=\frac{1}{2}\text{trace}(K_{y}K_{x}^{-1})+\frac{1}{2}\log|K_{x}|-\frac{1}{2}\log|K_{y}|-\frac{n}{2},$$

where |·| denotes the matrix determinant, *N*_*x *_and *N*_*y *_denote the zero-mean Gaussian distributions associated with *K*_*x *_and *K*_*y*_, respectively, and *n *is the number of samples. When the output kernel *K*_*y *_is defined as *K*_*y *_= **yy**^*T*^, the KL divergence in Eq. (9) can be expressed as

(10) $$\text{KL}(N_{y}|N_{x})=\frac{1}{2}y^{T}K_{x}^{-1}y+\frac{1}{2}\log|K_{x}|+\text{const},$$

where "const" denotes terms that are independent of *K*_*x*_, and *K*_*x *_is the input kernel matrix, which is defined as a linear combination of the given *p *kernels as

(11) $$K_{x}=\sum_{i=1}^{p}\alpha_{i}{\tilde{K}}_{i}+\lambda I=\sum_{i=1}^{p}\alpha_{i}\frac{e^{\beta_{i}L}}{\text{trace}(e^{\beta_{i}L})}+\lambda I.$$

Note that a regularization term, with λ as the regularization parameter, is added to Eq. (11) to deal with the singularity problem of kernel matrices as in [20], and we require $\sum_{i=1}^{p}\alpha_{i}=1$ as in multiple kernel learning (MKL) [17]. The optimal coefficients *α *= [*α*_1_, ..., *α*_*p*_]^*T *^are computed by minimizing KL(*N*_*y*_|*N*_*x*_). By substituting Eq. (11) into Eq. (10), and removing the constant term, we obtain the following optimization problem:

(12) $$\begin{array}{l} \underset{\alpha }{\min}\left\{a^{T}{\left(\sum_{i=1}^{p}\alpha_{i}{\tilde{K}}_{i}+\lambda I\right)}^{-1}a+\log\left|\sum_{i=1}^{p}\alpha_{i}{\tilde{K}}_{i}+\lambda I\right|\right\}\\ \begin{array}{cc} \text{s}\text{.t}\text{.} & \sum_{i=1}^{p}\alpha_{i}=1,\\ & \alpha \geq 0, \end{array} \end{array}$$

where *α *= (*α*_1_, ..., *α*_*p*_)^*T*^, *α *⩾ 0 denotes that all components of *α *are non-negative, and the vector *a *∈ ℝ^*n *^is the problem-specific target vector, corresponding to the general target in Eq. (9), defined as follows:

(13) $$a_{i}=\{\begin{array}{ll} 1 & \text{if }v_{i}\text{ is in the positive class},\\ -1 & \text{if }v_{i}\text{ is in the negative class},\\ 0 & \text{if }v_{i}\text{ is in the test set}. \end{array}$$

Note that we assign the label 0 to vertices in the test set so that it will not bias towards either class. Similar idea has been used in [24] for semi-supervised learning. In multiple kernel learning [17], the sum-to-one constraint on the weights is enforced as in Eq. (12). We present results on both constrained and unconstrained formulations in the experiments. Results show that the constrained formulations achieved better performance than the unconstrained ones.

Recall that the graph Laplacian matrix *L *is symmetric, so its eigen-decomposition can be expressed as

*L *= *PDP*^*T*^,

where

(14) *D *= diag(*d*_1_, ... ,*d*_*n*_)

is the diagonal matrix of eigenvalues and *P *∈ ℝ^*n *× *n *^is the orthogonal matrix of corresponding eigenvectors. According to the definition of the function of matrices [25], we have

(15) $$e^{\beta_{i}L}=PD_{i}P^{T},$$

where

(16) $$D_{i}=\text{diag}(e^{\beta_{i}d_{1}},\cdots ,e^{\beta_{i}d_{n}}).$$

The main result is summarized in the following theorem:

**Theorem 1**. *Given a set of p diffusion kernels, as defined in Eq. (7), the problem of learning the optimal kernel matrix, in the form of a convex combination of the given p kernel matrices as in Eq. (12), can be formulated as the following optimization problem:*

(17) $$\begin{array}{cc} \min_{\alpha } & \sum_{j=1}^{n}\left(\frac{b_{j}^{2}}{g_{j}}+\log(g_{j})\right) \end{array}$$

(18) $$\begin{array}{cc} subject\;to & \sum_{i=1}^{p}\alpha_{i}=1, \end{array}$$

(19) *α *≥ 0,

*where b *= (*b*_1_, ..., *b*_*n*_) = *P*^*T *^*a, g*_*j *_*is the j-th diagonal entry of the diagonal matrix G, defined as*

(20) $$G=\sum_{i=1}^{p}\alpha_{i}\frac{D_{i}}{trace(D_{i})}+\lambda I,$$

*and D*_*i *_*is the diagonal matrix defined in Eq.(16).*

*Proof*. The first term in Eq. (12) can be written as:

(21) $$\begin{array}{lll} a^{T}{\left(\sum_{i=1}^{p}\alpha_{i}{\tilde{K}}_{i}+\lambda I\right)}^{-1}a & = & a^{T}{\left(\sum_{i=1}^{p}\alpha_{i}\frac{e^{\beta_{i}L}}{\text{trace}(e^{\beta_{i}L})}+\lambda I\right)}^{-1}a\\ & = & a^{T}P{\left(\sum_{i=1}^{p}\alpha_{i}\frac{D_{i}}{\text{trace}(e^{\beta_{i}L})}+\lambda I\right)}^{-1}P^{T}a\\ & = & b^{T}{\left(\sum_{i=1}^{p}\alpha_{i}\frac{D_{i}}{\text{trace}(D_{i})}+\lambda I\right)}^{-1}b\\ & = & b^{T}G^{-1}b=\sum_{j=1}^{n}\frac{b_{j}^{2}}{g_{j}}, \end{array}$$

where the third equality follows from the property of the trace, that is,

$$\text{trace}(e^{\beta_{i}L})=\text{trace}(PD_{i}P^{T})=\text{trace}(D_{i}).$$

Similarly, the second term in Eq. (12) can be written as:

(22) $$\begin{array}{lll} \log\left|\sum_{i=1}^{p}\alpha_{i}{\tilde{K}}_{i}+\lambda I\right| & = & \log\left|\sum_{i=1}^{p}\alpha_{i}\frac{e^{\beta_{i}L}}{\text{trace}(e^{\beta_{i}L})}+\lambda I\right|\\ & = & \log\left|\sum_{i=1}^{p}\alpha_{i}\frac{e^{\beta_{i}D}}{\text{trace}(e^{\beta_{i}D})}+\lambda I\right|\\ & = & \log\left|G\right|\\ & = & \log(\prod_{j=1}^{n}g_{j})\\ & = & \sum_{j=1}^{n}\log(g_{j}). \end{array}$$

By combining the first term in Eq. (21) and the second term in Eq. (22), we prove the theorem.

The formulation in Theorem 1 is a nonlinear optimization problem. It involves a nonlinear objective function with *p *variables and linear equality and inequality constraints. Due to the presence of the log term in the objective, it is a non-convex problem and a globally optimal solution may not exist. However, our experimental results show that this formulation consistently produces superior performance.

#### Convex Formulation

The optimization problem in Theorem 1 is not convex. Previous studies [22,23] indicate that the removal of the log determinant term in the KL divergence criterion in Eq. (12) has a limited effect on the performance. This leads to the following optimization problem:

(23) $$\begin{array}{cc} \min_{\alpha } & a^{T}{\left(\sum_{j=1}^{n}\alpha_{i}{\tilde{K}}_{i}+\lambda I\right)}^{-1}a \end{array}$$

(24) $$\begin{array}{cc} \text{subject to} & \sum_{i=1}^{p}\alpha_{i}=1, \end{array}$$

(25) *α *≥ 0.

Following Theorem 1, we can show that the optimization problem above can be simplified as

(26) $$\begin{array}{cc} \min_{\alpha } & \sum_{j=1}^{n}\frac{b_{j}^{2}}{g_{j}} \end{array}$$

$$\begin{array}{cc} \text{subject to} & \sum_{i=1}^{p}\alpha_{i}=1, \end{array}$$

*α *≥ 0.

where *g*_*j *_and *b *are defined as in Theorem 1.

The optimization problem in Eq. (26) is convex and thus a globally optimal solution exists. Numerical experiments indicate that the simple gradient descent algorithm converges very quickly to the optimal solution. Furthermore, the prediction performance of this convex formulation is comparable to that of the formulation proposed in Theorem 1. This convex formulation shares some similarities with the one in [26], where a set of Laplacian matrices derived from multiple networks is combined.

### Diffusion Kernel Learning: The Multi-Task Case

It is known that proteins often perform multiple functions, which are typically related. Many existing function prediction approaches decouple multiple functions and formulate each function prediction problem as a separate binary-class classification problem. Such methods do not consider the relationship among the multiple functions of a protein and potentially compromise the overall performance.

We propose to extend our formulation for the single-task case to deal with multiple tasks simultaneously. In particular, we formulate a single optimization problem for the simultaneous prediction of multiple functions for a protein. The joint learning of multiple functions can potentially exploit the relationship among functions and improve the performance. In terms of computational complexity, the proposed joint optimization problem is shown to be comparable to that of the single-task formulation.

A key observation is that when the pre-specified diffusion kernels are computed from the same biological network with different values of *β*, the graph Laplacian matrices are the same for all tasks. By enforcing all tasks to share a common linear combination of kernels, we obtain the following joint optimization problem:

(27) $$\begin{array}{cc} \underset{\alpha }{\min} & \sum_{k=1}^{t}{\left(a^{\left(k\right)}\right)}^{T}{\left(\sum_{i=1}^{p}\alpha_{i}{\tilde{K}}_{i}+\lambda I\right)}^{-1}a^{\left(k\right)}+t\log\left|\sum_{i=1}^{p}\alpha_{i}{\tilde{K}}_{i}+\lambda I\right| \end{array}$$

(28) $$\begin{array}{cc} \text{subject to} & \sum_{i=1}^{p}\alpha_{i}=1, \end{array}$$

(29) *α *≥ 0,

where *a*^(*k*) ^∈ ℝ^*n *^for *i *= 1, ..., *t *is the vector of class labels for the *k*-th task as in Eq. (13), and *t *is the number of tasks. Note that all *t *tasks are related in this joint formulation by enforcing a common kernel matrix for all tasks. The objective function in Eq. (27) uses an equal weight for all tasks. If some tasks are known to be more important than others, a more general objective function with varying weights for different tasks may be used instead. Following Theorem 1, we can simplify the optimization problem in Eq. (27), as summarized in the following theorem:

**Theorem 2**. *Given a set of p diffusion kernels, as defined in Eq. (7), the problem of optimal multi-task kernel learning, in the form of a convex combination of the given p kernels, can be formulated as the following optimization problem:*

(30) $$\begin{array}{cc} \min_{\alpha } & \sum_{k=1}^{t}\sum_{j=1}^{n}\frac{b_{k}^{2}(j)}{g_{j}}+t \end{array}\sum_{j=1}^{n}\log(g_{j})$$

(31) $$\begin{array}{cc} subject\;to & \sum_{i=1}^{p}\alpha_{i}=1, \end{array}$$

(32) *α *≥ 0,

*where g*_*j *_*is defined as in Theorem 1, b*_*k *_= *P*^*T *^*a*^(*k*)^, *a*^(*k*) ^*is defined as in Eq. (13) for the k-th task, and t is the total number of tasks.*

*Proof*. The first term in Eq. (27) can be rewritten as

$$\begin{array}{lll} \sum_{k=1}^{t}\left(a^{{\left(k\right)}^{T}}{\left(\sum_{i=1}^{p}\alpha_{i}{\tilde{K}}_{i}+\lambda I\right)}^{-1}a^{\left(k\right)}\right) & = & \sum_{k=1}^{t}\left(b_{k}^{T}{\left(\sum_{i=1}^{p}\alpha_{i}\frac{D_{i}}{\text{trace}(D_{i})}+\lambda I\right)}^{-1}b_{k}\right)\\ & = & \sum_{k=1}^{t}(b_{k}^{T}G^{-1}b_{k})=\sum_{k=1}^{t}\sum_{j=1}^{n}\frac{b_{k}^{2}(j)}{g_{j}}. \end{array}$$

Similarly, the second term can be rewritten as

(33) $$t\log\left|\sum_{i=1}^{p}\alpha_{i}{\tilde{K}}_{i}+\lambda I\right|=t\sum_{j=1}^{n}\log(g_{j}).$$

The detailed intermediate steps of derivation are the same as those in the proof of Theorem 1 and thus are omitted. By combining these two terms together, we prove the theorem.

The optimization problem in Theorem 2 is not convex. Similar to the single-task case, the log determinant term in Eq. (27) may be removed, which leads to the following convex optimization problem:

(34) $$\begin{array}{cc} \min_{\alpha } & \sum_{k=1}^{t}\sum_{j=1}^{n}\frac{b_{k}^{2}(j)}{g_{j}} \end{array}$$

(35) $$\begin{array}{cc} \text{subject to} & \sum_{i=1}^{p}\alpha_{i}=1, \end{array}$$

(36) *α *≥ 0.

Experimental evidences show that this convex optimization problem is comparable to the formulation in Theorem 2 in prediction performance.

## Results and Discussion

We evaluate the performance of the proposed formulations on two benchmark data sets, and compare them with relevant methods, including the Neighbor Counting approach [4] and the FS-Weighted Averaging approach [5,6]. We construct 60 diffusion kernels from each data set using different values for *β *and the proposed formulations are applied to compute a linear combination of the pre-computed kernels. The performance of the obtained kernel is compared with that of the individual kernel. To see the relative performance of the objective functions, we also use the 1-norm soft margin SVM criterion, proposed in [17], to compute the linear combination of kernels and the results are presented. All of the formulations proposed in this paper are solved using the MATLAB [27] function *fmincon *which employs the sequential quadratic programming method [28]. The QCQP formulation for optimizing the 1-norm soft margin SVM criterion is solved using the MOSEK [29] software package. After the kernels are computed, they are fed into SVM for classification and the LIBSVM [30] software package is used in the experiments. All of the experiments are performed on a PC with Intel Pentium D 820 2.8G CPU and 2G RAM.

In the following experiments, a total of 60 diffusion kernels are pre-computed and the values of *β *used are *β*_*i *_= -0.1 × *i*, for *i *= 1, ..., 60. In order to investigate the performance of each individual kernel, we use each kernel for the classification and compute the average Receiver Operating Characteristic (ROC) values over all of the tasks. The ROC value produced by the best averaged individual kernel is used as a baseline. It is called rBaseline as all tasks are *restricted *to use the same kernel. We further relax the requirement that all tasks use the same kernel and compute the sequence of ROC values achieved by the best individual kernel for each of the tasks. This is considered another baseline called uBaseline as the kernel used by each task is *unrestricted*. Note that the kernel matrices for both rBaseline and uBaseline represent the single best candidate kernel in the ideal case that the labels of test data are known, and their performance is not guaranteed in practice. In contrast, the kernel matrices computed by the proposed formulations are the optimal kernel matrices in the form of linear combination of the given candidate kernel matrices. In order to evaluate the effectiveness of the weights obtained by the proposed formulations, we assign each kernel the same weight and compute the performance of the combined kernel. It is called eBaseline as all kernel matrices have an *equal *weight.

For convenience of presentation, the formulations proposed in Theorem 1, Eq. (26), Theorem 2, and Eq. (34) are denoted as DKL_KL_, DKL, mDKL_KL_, and mDKL, respectively. For DKL_KL _and mDKL_KL_, we also propose to remove the constraints in their optimization problems and the resulting formulations are denoted as ${\text{DKL}}_{\text{KL}}^{\text{u}}$ and ${\text{mDKL}}_{\text{KL}}^{\text{u}}$, respectively. (See the caption of Table 1 for detailed description.) The method based on optimizing 1-norm soft margin SVM criterion by solving QCQP proposed in [17] is denoted as SM1. The six proposed formulations are summarized in Table 1.

**Table 1:** Summary of the proposed formulations.${\text{DKL}}_{\text{KL}}^{\text{u}}$

Algorithm	Single task	Multiple tasks	Convexity	Constraint
DKL	✓		✓	✓
DKL_KL_	✓			✓
${\text{DKL}}_{\text{KL}}^{\text{u}}$	✓			
mDKL		✓	✓	✓
mDKL_KL_		✓		✓
${\text{mDKL}}_{\text{KL}}^{\text{u}}$		✓		

These formulations are categorized in terms of the number of tasks, convexity, and whether the weights on kernels are constrained. DKLKL, DKL, and ${\text{DKL}}_{\text{KL}}^{\text{u}}$ denote formulations using the Kullback-Leibler (KL) divergence criterion, the KL divergence criterion with the log term removed and the unconstrained version for single task, respectively. A lower case m is added before each method to denote the corresponding formulations for multiple tasks.

### Experiments on the Ligand Data Set

The Ligand data set was derived by Vert and Kanehisa [31] from the Ligand database of chemical reactions in biological pathways [32]. It contains a graph reflecting the interactions between proteins and the function information for them. The graph is a yeast biological network in which a path between vertices implies a possible series of reactions catalyzed by proteins along it. The numbers of vertices and edges in this graph are 753 and 7860, respectively. For the functions of proteins, the functional categories of the MIPS Comprehensive Yeast Genome Database (CYGD) [33] are considered as the gold standard. These categories are not mutually exclusive, and each protein may have multiple functions. There are 36 different functions considered for this data set.

#### Comparison of ROC Values

**Figure 1:**
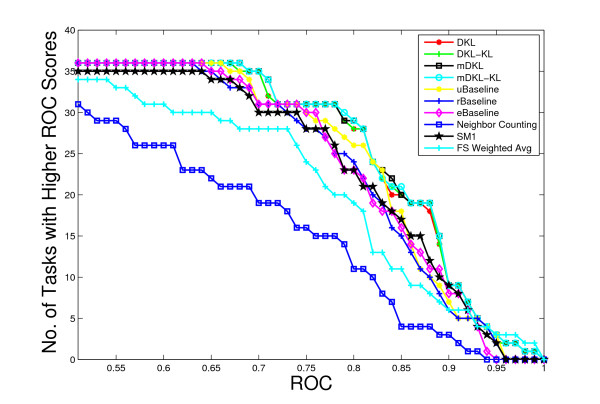
Comparison of ROC values for various algorithms on the Ligand Data Set. The horizontal axis represents the ROC values and the vertical axis is the number of tasks with ROC values above the corresponding horizontal axis value.

We use the ROC as the performance measure and the λ value is fixed to 10^-6 ^in the experiments. Our experimental results show that the algorithms are not sensitive to the value of λ, as long as it is neither too large nor too small. Figure 1 plots the number of tasks with ROC value above a threshold for all methods. The average ROC values achieved by all methods are also summarized in Table 2. In order to test statistical significance, we also compute the *p*-values of Wilcoxon signed test and the results are reported in Table 3. We can observe that mDKL achieves the best performance among all methods. All the proposed formulations except ${\text{mDKL}}_{\text{KL}}^{\text{u}}$ outperform the three baseline methods. This implies that the computed linear combination of kernels can potentially exploit the complementary information in different kernels and thus improve performance. The ROC value achieved by SM1 is lower than those of the three baseline methods, implying that the SVM criterion is less effective for such tasks. Note that the SM1 criterion also uses information from unlabeled data, but in a weak form. The ${\text{mDKL}}_{\text{KL}}^{\text{u}}$ formulation achieves a ROC value lower than the three baseline methods. This shows that the constraints have important normalizing effects and can not be removed. By comparing the relative performance of formulations with and without the log term, we can conclude that removing this term usually does not affect the performance. Another important observation is that mDKL and mDKL_KL _outperform DKL and DKL_KL_, implying that constraining the multiple tasks to share a common kernel does not degrade the performance if the kernel used is a linear combination of kernels obtained by the proposed formulations. In contrast, if the kernel used is a single kernel, this restriction will degrade the performance, as illustrated by the relative performance of rBaseline and uBaseline. For the eBaseline method, it can be observed that, except for ${\text{mDKL}}_{\text{KL}}^{\text{u}}$, all of other proposed formulations outperform it. This illustrates that our formulations can compute an optimal kernel matrix by assigning different weights to the candidate kernel matrices. We can observe from Table 3 that the difference between the performance of the two baseline methods (rBaseline and eBaseline) and that of DKL and mDKL are statistically significant. All diffusion kernel based approaches are competitive with the Neighbor Counting approach [4] and the FS-Weighted Averaging approach [5,6]. Neighbor Counting and FS-Weighted Averaging use the local information, more specifically the level-1 neighborhood (Neighbor Counting) and both level-1 and level-2 neighborhoods (FS-Weighted Averaging), for the prediction. The experimental results show the effectiveness of capturing the long-range relationships (global information) between proteins in the network in diffusion kernels [15].

**Table 2:** Mean ROC values and execution time (in seconds) of various methods on the Ligand Data Set.

Algorithm	Mean ROC	Time
uBaseline	0.8346	----
rBaseline	0.8223	----
eBaseline	0.8267	----
DKL	0.8523	165.52
DKL_KL_	0.8523	665.13
${\text{DKL}}_{\text{KL}}^{\text{u}}$	0.8365	4406.64
mDKL	0.8542	5.61
mDKL_KL_	0.8538	12.33
${\text{mDKL}}_{\text{KL}}^{\text{u}}$	0.8213	62.80
Neighbor Counting	0.7010	----
FS-Weighted Averaging	0.7785	----
SM1	0.8162	739.69

rBaseline denotes the baseline method that requires all tasks share a common kernel, uBaseline corresponds to the baseline method without this requirement, and eBaseline denotes the baseline in which all of the candidate kernel matrices are assigned the same weight.

**Table 3:** *p*-values obtained from Wilcoxon signed test comparing DKL and mDKL with other formulations for the Ligand data set.

Algorithm	DKL	mDKL
uBaseline	1.456E-2	5.690E-3
rBaseline	8.188E-4	2.091E-4
eBaseline	7.524E-5	2.548E-5
DKL	1.000	1.249E-2
DKL_KL_	3.217E-1	1.279E-2
${\text{DKL}}_{\text{KL}}^{\text{u}}$	1.112E-4	3.852E-5
mDKL	1.249E-2	1.000
mDKL_KL_	1.404E-1	6.849E-2
${\text{mDKL}}_{\text{KL}}^{\text{u}}$	3.852E-5	7.013E-6

For the Ligand data set, each algorithm produces an ROC vector consisting of ROC values over each task. We use Wilcoxon signed test to test the difference of the paired data. The null hypothesis of this test is that the ROC vectors produced by the two compared algorithms have the common median. The numbers reported in this table are the *p*-values that represent the probabilities that the null hypothesis is true. Typically, the null hypothesis is rejected if *p *< 0.05.

**Figure 2:**
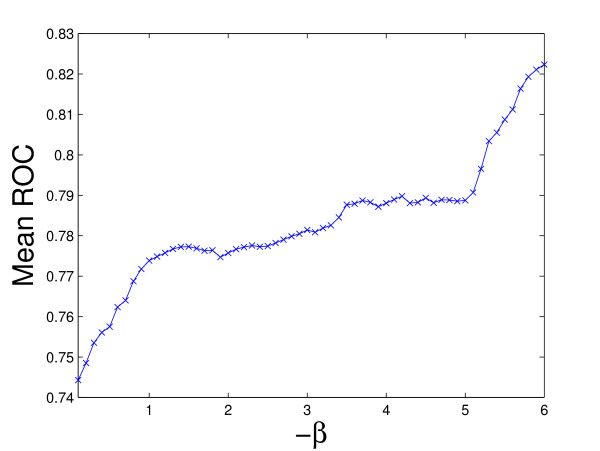
Mean ROC values over 36 tasks for each kernel on the Ligand Data Set (the kernel with the maximum mean ROC value is used in rBaseline). The horizontal axis denotes the -*β *values used to build the corresponding kernel and the vertical axis is the mean ROC value.

**Figure 3:**
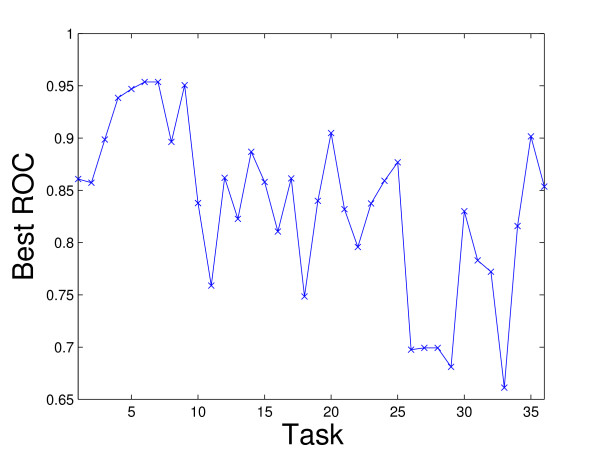
Best ROC values for tasks achieved by the best kernel (uBaseline) on the Ligand Data Set. The horizontal axis represents the tasks and the vertical axis is the corresponding best ROC value.

**Figure 4:**
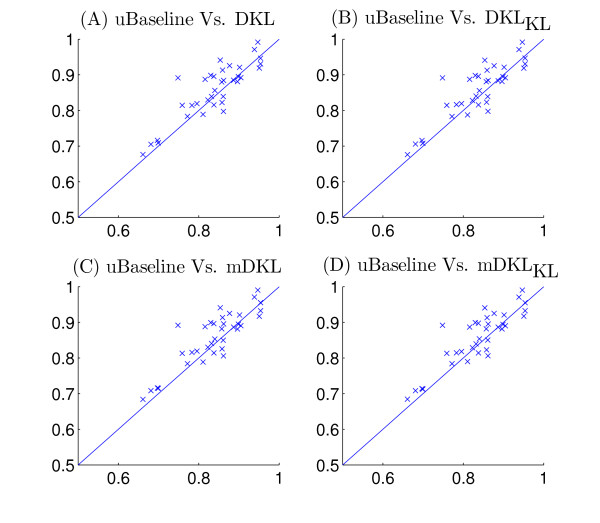
Comparison of the relative performance of the proposed formulations with that of uBaseline on the Ligand Data Set. The horizontal axis represents uBaseline and the vertical axis corresponds to DKL, DKL_KL_, mDKL, mDKL_KL_. Each point in the scatter plots corresponds to ROC values produced by the compared methods on the same task.

Figure 2 plots the average ROC values for the 60 kernels (the maximum mean ROC value is used in rBaseline) and Figure 3 plots the best ROC values for the 36 tasks. We can observe that for tasks 29 and 33, the best ROC values are small. This implies that all the kernels perform poorly for these two tasks. To illustrate the relative performance of the proposed formulations with that of the baseline method graphically, we plot in Figure 4 the ROC values obtained by the proposed formulations with respect to uBaseline using scatter plots. We can observe that there are two points below the 45-degree line in each plot. Those two points correspond to tasks 29 and 33 and they are difficult to classify by all methods. As most points in the plots are above the 45-degree line, we can conclude that the proposed formulations outperform uBasline on most tasks.

#### Comparison of Execution Time

In order to compare the efficiency of various kernel learning methods, we list in Table 2 the execution time of the compared methods. It can be observed that all methods based on multiple tasks are more efficient than their single-task counterparts. In particular, the execution time of mDKL is roughly 1/36 of that of DKL, which is consistent with our theoretical analysis. In general, convex formulations are more efficient than their non-convex original formulations and the optimization problems with the constraints removed take a longer time to converge. By taking the performance into account, the DKL and mDKL may be the best choices in practice.

#### Stability Test

In order to obtain a robust performance estimate for the various methods, we randomly partition the data set into a training set and a test set ten times and the average ROC values and standard deviations across splittings are reported in Table 4. Compared with the results in Table 2, we can see that the relative performance of each method in these two tables is very similar. In particular, mDKL and mDKL_KL _achieve the best overall performance. Except for the two unconstrained formulations ${\text{DKL}}_{\text{KL}}^{\text{u}}$ and ${\text{mDKL}}_{\text{KL}}^{\text{u}}$, all of other proposed formulations achieve higher ROC values than the three baseline methods. It is worth noting that the performance of uBaseline and rBaseline is obtained by using the labels of both the training and test data and such performance is not guaranteed in practice when only the labels of the training data are used.

**Table 4:** Average ROC values and the corresponding standard deviations over 11 splittings on the Ligand Data Set. One of the splittings was specified by the contributor of the data and the remaining ten splittings are randomly generated.

Algorithm	Average ROC	Standard deviation
uBaseline	0.8326	0.0064
rBaseline	0.8181	0.0062
eBaseline	0.8193	0.0070
DKL	0.8493	0.0034
DKL_KL_	0.8493	0.0032
${\text{DKL}}_{\text{KL}}^{\text{u}}$	0.8318	0.0061
MDKL	0.8507	0.0033
mDKL_KL_	0.8507	0.0033
${\text{mDKL}}_{\text{KL}}^{\text{u}}$	0.8209	0.0031

### Experiments on the von Mering Data Set

**Figure 5:**
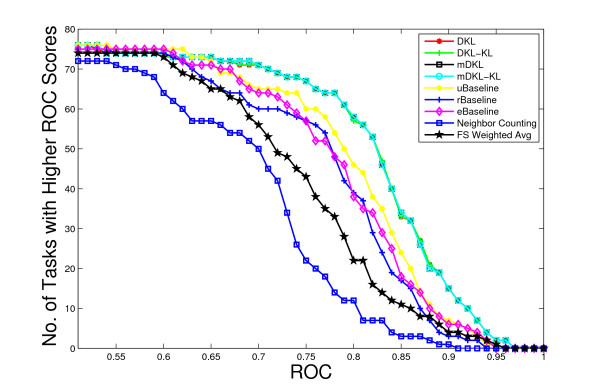
Comparison of ROC values for various algorithms on the von Mering Data Set. The horizontal axis represents the ROC values and the vertical axis is the number of tasks with ROC values above the corresponding horizontal axis value.

**Figure 6:**
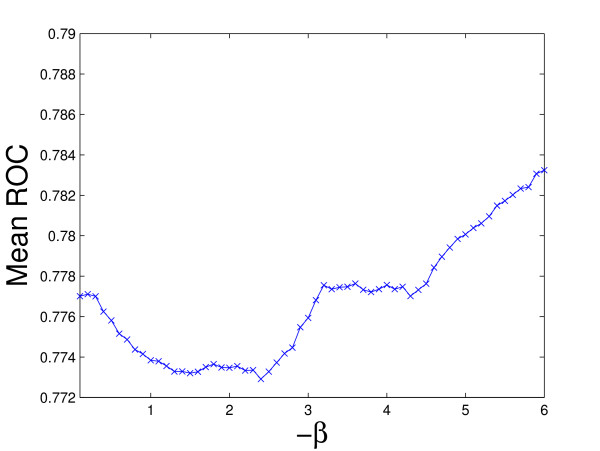
Mean ROC values over 76 tasks for each kernel on the von Mering Data Set. The horizontal axis denotes the -*β *values used to build the corresponding kernel and the vertical axis is the mean ROC values.

**Figure 7:**
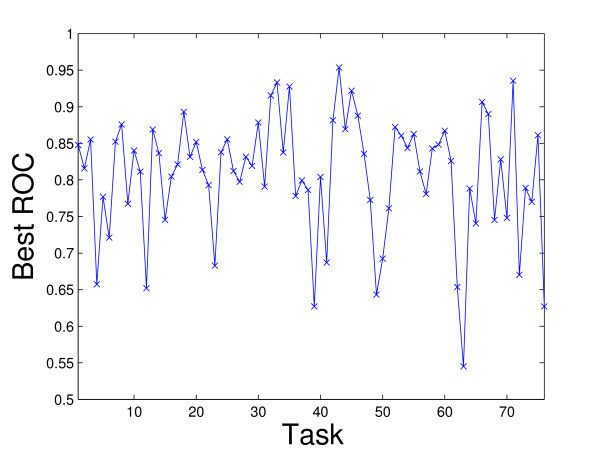
Best ROC values for diferent tasks achieved by different kernels on the von Mering Data Set. The horizontal axis represents the tasks and the vertical axis is the corresponding best ROC values.

**Figure 8:**
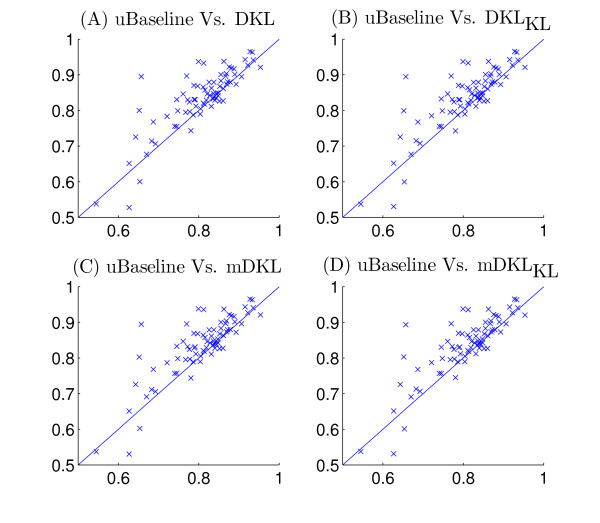
Comparison of the relative performance of the proposed formulations with that of uBaseline on the von Mering Data Set. The horizontal axis represents uBaseline and the vertical axis corresponds to DKL, DKL_KL_, mDKL, mDKL_KL_. Each point in the scatter plots corresponds to ROC values produced by the compared methods on the same task.

The von Mering data set was created by von Mering *et al*. [34] from protein-protein interactions identified via six different methods. It contains a graph consisting of 2617 vertices (proteins) and 11855 edges. There are 76 different functions (tasks) associated with the proteins in the graph. The performance of different methods is reported in Figure 5. Two baseline methods, rBaseline and uBaseline, constructed exactly the same way as those for the Ligand data set are used and their performance is summarized in Figure 6 and Figure 7, respectively. The value for is again set to 10^-6 ^in the experiments. Figure 8 compares the relative performance of the proposed formulations with that of the uBaseline graphically.

#### Comparison of ROC Values

We use the ROC values of each method to compare their relative performance. Similar to Figure 1 for the Ligand data set, Figure 5 plots the change of the number of tasks with ROC value above a certain threshold as the threshold varies for each of the compared method. For ease of comparison, Table 5 also lists the average ROC values achieved by the compared methods. Similarly, the *p*-values of Wilcoxon signed test for this data set are reported in Table 6. As the SM1 formulation requires excessive storage and computational time for this relatively large data set, we are not able to obtain its result in this experiment. From these results we can observe that mDKL and mDKL_KL _achieve the best performance. In general, the performance of DKL, DKL_KL_, mDKL, and mDKL_KL _is very close. All of the proposed formulations except ${\text{DKL}}_{\text{KL}}^{\text{u}}$ perform better than the three baseline methods. The difference between DKL and DKL_KL _as well as the difference between mDKL and mDKL_KL _is very small, which further confirms that the removal of the log term does not affect the performance of algorithm much. For the formulations with constraints removed, i.e., ${\text{DKL}}_{\text{KL}}^{\text{u}}$ and ${\text{mDKL}}_{\text{KL}}^{\text{u}}$, their performance is the lowest among the proposed formulations. Similar to the case for the Ligand data set, we conclude that constraining the multiple tasks to share a common kernel does not degrade the performance if the kernel used is a linear combination of kernels obtained by the proposed formulations. In contrast, if the kernel used is a single kernel, this restriction will degrade the performance, as illustrated by the relative performance of rBaseline and uBaseline. In terms of the eBaseline, we can observe from Table 5 that all of our proposed formulations achieve higher ROC values than the eBaseline method, in which all of the kernel matrices are assigned the same weight. We can observe from Table 6 that the difference between the performance of all of the three baselines and that of DKL and mDKL is statistically significant. We can again observe that all diffusion kernel based approaches are competitive with the Neighbor Counting approach and the FS-Weighted Averaging approach.

**Table 5:** Mean ROC values and execution time (in seconds) of various methods on the von Mering Data Set.

Algorithm	Mean ROC	Time
uBaseline	0.8061	----
rBaseline	0.7832	----
eBaseline	0.7945	----
DKL	0.8339	3441.13
DKL_KL_	0.8340	7445.67
${\text{DKL}}_{\text{KL}}^{\text{u}}$	0.7968	35384.84
mDKL	0.8345	54.64
mDKL_KL_	0.8345	67.52
${\text{mDKL}}_{\text{KL}}^{\text{u}}$	0.8129	151.49
Neighbor Counting	0.7076	----
FS-Weighted Averaging	0.7544	----
SM1	----	----

**Table 6:** *p*-values obtained from Wilcoxon signed test comparing DKL and mDKL with other formulations for the von Mering data set.

Algorithm	DKL	mDKL
uBaseline	5.849E-7	2.966E-7
rBaseline	1.510E-10	1.038E-10
eBaseline	4.268E-10	2.863E-10
DKL	1.000	6.531E-2
DKL_KL_	3.776E-1	1.669E-1
${\text{DKL}}_{\text{KL}}^{\text{u}}$	6.870E-11	3.193E-11
mDKL	6.531E-2	1.000
mDKL_KL_	5.971E-2	1.637E-1
${\text{mDKL}}_{\text{KL}}^{\text{u}}$	3.193E-11	8.252E-12

For the von Mering data set, each algorithm produces an ROC vector consisting of ROC values over each task. We use Wilcoxon signed test to test the difference of the paired data. The null hypothesis of this test is that the ROC vectors produced by the two compared algorithms have the common median. The numbers reported in this table are the *p*-values that represent the probabilities that the null hypothesis is true. Typically, the null hypothesis is rejected if *p *< 0.05.

Figure 8 presents the scatter plots of four proposed formulations with respect to uBaseline. It can be observed that most points are above the 45-degree line, which implies that the linear combination of kernels is better than the ideally best individual kernel. In general, the performance of DKL_KL_, DKL, mDKL_KL_, mDKL is better than uBaseline. And this is also confirmed by the mean ROC values listed in Table 5.

#### Comparison of Execution Time

Table 5 also lists the execution time of various kernel learning methods. Similar conclusions can be drawn from this table as to the execution time on the Ligand data set. All methods based on multiple tasks are more efficient than their single-task counterparts. By comparing the results in Table 2 and Table 5 we can also observe that as the number of tasks increases, the time difference between methods based on multiple tasks and those based on single tasks increases too. Thus, the formulations based on multiple tasks are preferred when the number of tasks is large.

#### Stability Test

Similar to the Ligand data set, we generate ten random splittings of the data into training and test sets and report the average ROC values and standard deviations in Table 7. By comparing with results in Table 5, we can see that the relative performance of each method is similar in both tables. All of the proposed formulations outperform eBaseline.

**Table 7:** Average ROC values and the corresponding standard deviations over 11 splittings on the von Mering Data Set. One of the splittings was specified by the contributor of the data and the remaining ten splittings are randomly generated.

Algorithm	Average ROC	Standard deviation
uBaseline	0.8078	0.0045
rBaseline	0.7863	0.0064
eBaseline	0.7909	0.0078
DKL	0.8398	0.0042
DKL_KL_	0.8398	0.0042
${\text{DKL}}_{\text{KL}}^{\text{u}}$	0.7991	0.0059
mDKL	0.8402	0.0042
mDKL_KL_	0.8402	0.0042
${\text{mDKL}}_{\text{KL}}^{\text{u}}$	0.8194	0.0046

## Conclusion

In this paper, we address the issue of learning an optimal diffusion kernel based on KL divergence criterion for protein function prediction. By exploiting the special structure of the diffusion kernel, we show that this KL divergence based kernel learning problem can be formulated as a simple optimization problem, which can be solved efficiently. We also extend the formulation to the multi-task case where we predict multiple functions of a protein simultaneously.

We have conducted experiments on two benchmark data sets. Our results show that the performance of linearly combined diffusion kernel is better than every single candidate diffusion kernel. Results also show that the removal of the log term in the KL divergence criterion does not degrade its recognition performance, while it leads to a reduced computational cost. When the number of tasks is large, the algorithms based on multiple tasks are favored due to their competitive recognition performance and small computational costs. One possible extension is to incorporate the learning of the regularization parameter in the proposed formulations as in [17]. The difference between the proposed learning framework and those in [17] is that our formulations require that the eigenvectors of the candidate kernel matrices to be the same. Thus the proposed formulations may not be applied for heterogeneous data integration. We plan to apply the proposed algorithms for the analysis of other graph-based biological data.

## Authors' contributions

LS designed the methodology, implemented programs, and participated in manuscript preparation. SJ derived the KL divergence formulation, and drafted the manuscript. JY originally conceived the project, guided the implementation, and drafted the manuscript. All authors have read and approved the final manuscript.
